# Lateral wedges with and without custom arch support for people with medial knee osteoarthritis and pronated feet: an exploratory randomized crossover study

**DOI:** 10.1186/s13047-017-0201-x

**Published:** 2017-05-02

**Authors:** Michael A. Hunt, Judit Takacs, Natasha M. Krowchuk, Gillian L. Hatfield, Rana S. Hinman, Ryan Chang

**Affiliations:** 1 Department of Physical Therapy, University of British Columbia, 212-2177 Wesbrook Mall, Vancouver, BC V6T 1Z3 Canada; 2 Department of Physiotherapy, University of Melbourne, Melbourne, VIC Australia; 3 Human Performance Engineering Laboratory, Reebok International, Canton, MA USA

**Keywords:** Knee, Osteoarthritis, Lateral wedges, Pain, Function

## Abstract

**Background:**

Pronated foot posture is associated with many clinical and biomechanical outcomes unique to medial compartment knee osteoarthritis (OA). Though shoe-worn insole treatment, including lateral wedges, is commonly studied in this patient population, their effects on the specific subgroup of people with medial knee OA and concomitant pronated feet are unknown. The purpose of this study was to evaluate whether lateral wedge insoles with custom arch support are more beneficial than lateral wedge insoles alone for knee and foot symptoms in people with medial tibiofemoral knee osteoarthritis (OA) and pronated feet.

**Methods:**

Twenty-six people with pronated feet and symptomatic medial knee OA participated in a randomized crossover study comparing five degree lateral wedge foot insoles with and without custom foot arch support. Each intervention was worn for two months, separated by a two-month washout period of no insoles wear. Main outcomes included the Western Ontario and McMaster Universities Osteoarthritis Index (WOMAC) pain and physical function subscales, the revised short-form Foot Function Index (FFI-R) pain and stiffness subscales, and the timed stair climb test. Regression modeling was conducted to examine treatment, period, and interaction effects.

**Results:**

Twenty-two participants completed the study, and no carryover or interaction effects were observed for any outcome. Significant treatment effects were observed for the timed stair climb, with greater improvements seen with the lateral wedges with arch support. Within-condition significant improvements were observed for WOMAC pain and physical function, as well as FFI-R pain and stiffness with lateral wedges with arch support use. More adverse effects were reported with the lateral wedges alone, while more people preferred the lateral wedges with arch support overall.

**Conclusions:**

Addition of custom arch support to a standard lateral wedge insole may improve foot and knee symptoms in people with knee OA and concomitant pronated feet. These preliminary findings suggest further research evaluating the role of shoe-worn insoles for treatment of this specific sub-group of people with knee OA is warranted.

**Trial registration:**

Clinicaltrials.gov identifier: NCT02234895.

## Background

Osteoarthritis (OA), commonly affecting the knee joint, is one of the most prevalent chronic musculoskeletal disorders and is a leading cause of long-term physical disability affecting adults [1]. While we are unaware of joint specific indicators of the economic burden of OA, a recent report indicates that more than 6.9% of the adult population in the Unites States had symptomatic knee OA in 2007-2008 [2] – a number that is expected to rise dramatically in the coming decades. Further, symptomatic OA (the combination of symptoms and radiographic evidence of OA) is more commonly found in the knee than in any other joint [3]. Given that there is no cure for knee OA and the overall economic burden of OA is high, there has been a recent push towards the identification of non-surgical, non-pharmacological treatments for knee OA that can be delivered effectively, safely, and with minimal personnel and economic resources [4]. Shoe-worn insoles/orthotics are a low-cost and low-burden self-management option that has widespread appeal for managing knee OA symptoms.

Recent research confirms the link between knee and foot problems in people with knee OA. An examination of data from the Osteoarthritis Initiative showed that 25% of individuals with painful knee OA concurrently report foot pain, and that the presence of foot pain adversely affected overall health and function [5]. Individuals with knee OA also tend to exhibit more pronated feet compared to those without knee OA [6, 7]. Indeed, a recent study involving 164 people with symptomatic medial tibiofemoral OA reported that 45% had pronated or severely pronated feet [8]. Recent research has also shown that the presence of foot/ankle symptoms significantly increases the odds of developing knee OA symptoms and symptomatic radiographic knee OA [9]. Additionally, rearfoot eversion during walking has also been shown to be associated with medial compartment knee joint load, as quantified by the external knee adduction moment (KAM) [10]. Specifically, more rearfoot eversion appears to be associated with lower KAM values. Finally, older adults with pronated feet are more likely to exhibit knee pain and medial tibiofemoral cartilage damage than older adults with other foot types [11]. Taken together, these findings indicate that people with pronated feet form a large, and clinically relevant, sub-group of the population with knee OA. Thus, targeted treatment approaches for this subgroup that considers their unique biomechanical needs may be warranted. However, current methods for the treatment of knee OA symptoms and biomechanics has typically failed to directly address any aspect of foot biomechanics in general, and in those with knee OA and concomitant pronated feet specifically.

A commonly studied conservative treatment approach for knee OA is shoe-worn insoles, in particular insoles that are built up along the lateral edge (lateral wedges). There have been a number of studies examining the biomechanical and clinical changes associated with use of lateral wedges. Although lateral wedges have been shown to provide immediate reductions in KAM magnitudes [12, 13] – consistent with the reported negative correlation between increased rearfoot eversion (which would occur with lateral wedging) and the KAM [10] – the effects on knee symptoms are less clear [14]. A primary limitation of previous lateral wedges for knee OA research has been a failure to consider participants’ foot morphology in the study design and delivery of treatment. In clinical practice, pronated feet are typically managed with some form of medial arch support [15, 16], and while some research shows that beneficial changes in ankle kinematics [17] and foot pain [18, 19] may be obtained with this clinical approach in people with pronated feet, the evidence in this area is sparse [20]. However, there is potential that a combination of lateral wedges and arch supports may be beneficial for people with painful knee OA and pronated feet [21].

Given the apparent strong link between pronated foot type and disease characteristics specific to medial compartment knee OA, and that biomechanical interventions such as lateral wedges are advocated in clinical guidelines for the use of conservative treatments for knee OA in general [22], a better understanding of the clinical effects of shoe-worn insole treatment for in this specific subgroup of knee OA is warranted. Therefore, the purpose of the present exploratory randomized crossover study was to compare clinical effects (knee and foot symptoms) of lateral wedges alone to insoles that combined lateral wedging with customized arch support in people with medial knee OA and concomitant pronated feet. We were also interested in the safety of these interventions and adherence to treatment. It was hypothesized that the combined insole would produce greater improvements in knee pain and physical function, as well as foot pain and stiffness, and would be more comfortable.

## Methods

### Participants

Community-dwelling individuals with medial tibiofemoral OA were recruited via advertisements in print media. Inclusion criteria included: (i) definitive medial tibiofemoral osteophytes on radiograph; (ii) joint space narrowing greater in the medial tibiofemoral compartment compared to the lateral compartment; (iii) history of knee pain longer than six months; (iv) average knee pain of at least 3 out of 10 (using an 11-point numerical rating scale (NRS) with terminal descriptors of 0 = “no pain” and 10 = “worst pain imaginable”) over the one month prior to initial screening; and (v) pronated feet, defined as +4 or higher on the Foot Posture Index [23], with at least four items rated as +1 (indicating pronation). Exclusion criteria included: (i) knee surgery or intra-articular injection for pain within the previous six months; (ii) current or past (within six weeks) oral corticosteroid use; (iii) history of knee joint replacement or tibial osteotomy; (iv) any other condition affecting lower limb function; and (v) current, or failed previous (i.e. premature cessation of orthotic treatment due to adverse effects), wearing of shoe insoles/orthotics. The study was approved by the Institution’s Clinical Research Ethics Board and all participants provided written informed consent. In contrast to information provided on the trials registry (Clinicaltrials.gov identifier: NCT02234895), gait biomechanics data were only collected at the initial baseline data collection session, rather than at all time points, and are presented elsewhere [24].

### Study design

This was an exploratory, within-subjects cross-over study.

### Procedures

Interested participants were initially screened for inclusion and exclusion criteria, and eligible individuals were referred for a physical screening of foot posture. Individuals who passed the foot posture eligibility criteria described above were referred for radiographic evaluation. Standing, semi-flexed, postero-anterior radiographs were obtained and graded for disease severity using the Kellgren and Lawrence (KL) OA classification system [25]. Individuals who met all study eligibility criteria were then referred to a Canadian Certified Pedorthist, who obtained foot measurements and made volumetric casts of both feet to inform manufacture of insoles. Participants returned to the same Pedorthist following manufacture of both sets of the insoles for final modifications and instruction on insoles wear. Insoles were made for bilateral wear, even in cases where knee OA was unilateral. Both sets of insoles were then sent directly to the University laboratory, and participants were scheduled for their first baseline testing session.

Following baseline assessment of outcomes (month 0), participants were randomly allocated to either i) lateral wedges alone or; ii) lateral wedges plus custom arch support. Allocated insoles were worn for a two-month period in their own usual shoes, and then participants were re-assessed (month 2). After a two-month washout period of no insole wear, participants were re-assessed again (month 4) and then underwent a second two-month period of intervention with the alternate insole condition, after which they were re-assessed for the final time (month 6). The randomization schedule was produced before study commencement by a researcher not involved with data collection or analysis. Random permuted blocks of 4 or 6 were developed and randomization codes were sealed in consecutively numbered, opaque envelopes that were opened after baseline testing for each participant. To prevent cross-contamination of insole wear, the unused pair (in the case of active treatment periods), or both pairs (in the case of the wash-out period) of insoles were kept on-site at the University. Further, the participants were unaware of the study hypotheses as well as the justification for the design of the insoles. Both pairs were provided to participants upon study completion.

### Interventions

**Fig. 1:**
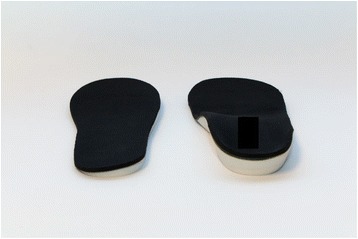
Intervention insoles. Left: the lateral wedge only condition consisted of a sulcus-length 5-degree lateral wedge. Right: the lateral wedge plus custom foot arch support involved the 5-degree sulcus-length lateral wedge built into a negative of the volumetric cast that provided the support across the entire foot

For both types of insoles, polypropylene sheets of 3-4 mm thickness were vacuum formed or milled directly to produce a sulcus length insole. For both conditions, the lateral wedges consisted of a 5-degree ethyl-vinyl-acetone (EVA) (Shore A hardness of 55) sulcus-length posting. For the lateral wedges plus arch support insole, the same lateral wedges were used in combination with a custom arch support across the foot to match the negative of the volumetric cast. Both insoles (Fig. 1) were finished with the same neoprene cover and modified as necessary to maximize comfort and fit. Participants were instructed to wear the insoles as much as possible and no other instructions regarding the intervention were provided.

### Outcome measurement

Outcome assessments were conducted by the same blinded assessor. Testing sessions at the University included completion of self-report questionnaires and objective assessment of physical function.

Self-reported knee and foot symptoms were the clinical outcomes of interest. Participants completed the Western Ontario and McMaster Universities Arthritis Index (WOMAC) Likert version, from which the pain and physical function subscales were calculated [26]. The WOMAC contains 24 items that quantify pain (5 items), stiffness (2 items), and physical function (17 items) that has been used in numerous knee OA studies. Foot symptoms were assessed using the Foot Function Index (revised - short form) (FFI-R). This self-report questionnaire consists of 34 items that provide the ability to quantify aspects of foot pain, disability and activity limitation [27]. For the purposes of this study, the pain and stiffness subscales were used. Each question is rated from 1 (no pain or stiffness) to 4 (severe pain or stiffness). Given that “not applicable” is a potential response for some questions, questions rated as “not applicable” were removed and the remaining scores within that subscale were summed. Subscale scores were then converted to a percentage of the maximum potential score for that particular subscale with 25% indicating no pain or stiffness and 100% indicating severe pain or stiffness.

Objective physical function was assessed using the timed stair climb test, where participants were instructed to ascend 12 stairs “as quickly as possible” and the fastest time from two attempts was recorded [28]. Adherence to each insole intervention was self-reported in daily log books. Participants reported the total number of hours of shoe wear each day as well as the total number of hours wearing the insole. Wear time was then calculated by the research team following the intervention, and expressed as total number of insole wear hours, as well as a percentage of total shoe wear time. Participants also recorded perceived adverse effects of the insoles, and changes in treatment regimens during the active treatment periods (months 0 to 2 and 4 to 6) in their daily log books. At the month 2 and month 6 assessments, participants rated comfort during the intervention using an 11-point NRS with terminal descriptors of 0 = “maximum discomfort” and 10 = “maximum comfort”. Participants also rated their perceived overall change in symptoms at follow-up (month 2 and 6) compared to baseline (month 0 and 4) using a 5-point Likert scale (1 = “much worse”, 2 = “slightly worse”, 3 = “no change”, 4 = “slightly better”, and 5 = “much better”). Finally, at the month 6 assessment, participants indicated which pair of insoles (“first or second”) they preferred.

### Statistical analysis

All statistical analyses were conducted using Statistical Analysis Software (SAS, v 9.4; SAS Institute Inc.: Cary, NC, USA) and p values <0.05 were considered significant. For WOMAC, FFI-R and timed stair climb outcomes, change scores were calculated as follow-up minus baseline values for each intervention period. As comfort data were only acquired at the follow-up assessments, these were treated as raw data rather than change scores. Mixed effects regression models predicting change variables against fixed effects for intervention period and treatment type, and a random subject effect were developed for each outcome variable. Interactions between intervention period and treatment type were initially included as predictors in a second model, then dropped from the models if non-significant (this was true for all models).

## Results

Between July 2014 and June 2015, 127 individuals underwent eligibility screening, of which 26 were enrolled. Participant demographics are summarized in Table 1. Fourteen individuals were randomized to receive the lateral wedge plus arch support in the first iteration (months 0-2), while 12 participants received the lateral wedges alone in the first iteration. Twenty-four individuals completed their first allocated treatment period, and of those, 22 completed month 6 assessment. Of the four participants who did not complete the study, three were lost-to-follow-up (one prior to the month 2 follow-up assessment, one after the month 4 baseline assessment, and one prior to the month 6 follow-up assessment), while the fourth withdrew prior to the month 2 follow-up assessment due to health issues unrelated to the study. Mean (sd) intervention times were 2.2 (0.2) months for the lateral wedges and 2.1 (0.2) months for the lateral wedges plus arch support, with a washout period of 2.1 (0.3) months.

**Table 1:** Participant characteristics at baseline based on order of intervention. Values are mean (sd) unless otherwise noted

	Wedge to Wedge plus arch support (*n* = 12)	Wedge plus arch support to Wedge (*n* = 14)	All participants (*n* = 26)
Age (years)	66.3 (10.2)	62.1 (5.0)	64.0 (7.6)
Sex (F:M)	9:3	13:1	22:4
Foot posture^a^	5 (4,8)	5 (4,9)	5 (4,9)
Body mass (kg)	65.3 (9.8)	75.0 (15.3)	70.5 (13.8)
BMI (kg/m^2^)	25.2 (3.0)	28.9 (4.5)	27.2 (4.2)
Disease severity (n)			
KL 2	9	7	16
KL 3	3	7	10

^a^ As quantified by the foot posture index (FPI). Values are reported as median (minimum, maximum)

**Fig. 2:**
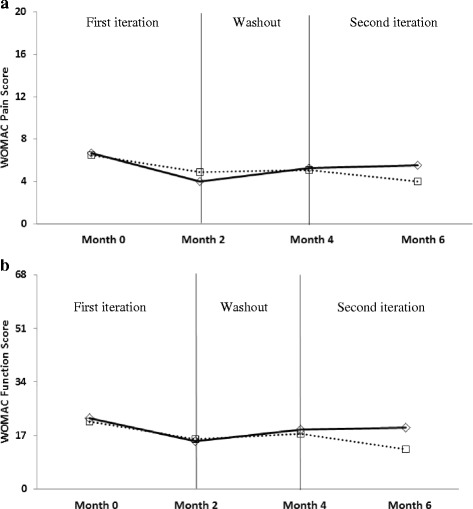
Assessment of treatment and period effects for Western Ontario and McMaster Universities Osteoarthritis Index (WOMAC) pain **a** and physical function subscales **b**. Lower scores indicate less knee pain and physical dysfunction. Dotted lines indicate mean values for participants receiving the lateral wedges alone first, followed by the lateral wedges plus arch support; solid lines indicate mean values for participants receiving the lateral wedges plus arch support first, followed by the lateral wedges alone

**Fig. 3:**
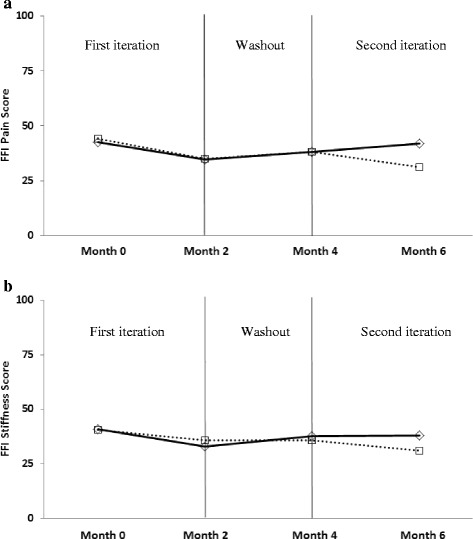
Assessment of treatment and period effects for Foot Function Index (FFI-R) pain **a** and stiffness subscales **b**. Values are presented as percentages of the maximum scores for each subscale, and lower scores indicate less foot pain and foot stiffness. Dotted lines indicate mean values for participants receiving the lateral wedges alone first, followed by the lateral wedges plus arch support; solid lines indicate mean values for participants receiving the lateral wedges plus arch support first, followed by the lateral wedges alone

Table 2 provides results for clinical outcomes across test conditions. The lateral wedges plus arch support resulted in significant improvements in all parameters, whilst the lateral wedges alone did not significantly change any clinical outcome. However, these differences generally did not translate into significant treatment main effects (between conditions), with the exception of timed stair climb, where improvements were significantly greater with the use of the lateral wedges with arch support compared to lateral wedges alone (*p* = 0.02). In addition, a significant period effect (Figs. 2 and 3) was seen for WOMAC Pain scores (*p* = 0.04). There were no intervention by period interactions.

**Table 2:** Outcomes data. Mean (sd) baseline and follow-up outcome data for all variables, as well as model adjusted differences (follow-up minus baseline; difference (95% confidence intervals)) within each condition as well as between conditions. Model estimate values represent the within- and between-condition least-square mean differences adjusted for period and random subject effects from the regression modeling

	Lateral wedges (*n* = 24)			Lateral Wedges plus Arch Support (*n* = 22)			Between-conditions comparison
Variable	Baseline	Follow-up	Difference	Baseline	Follow-up	Difference	Difference
WOMAC Pain (0-20)	5.9 (3.0)	5.2 (2.9)	-0.7 (-1.8, 0.5)	6.3 (3.4)	4.0 (2.5)	-1.9 (-3.1, -0.6)*	-1.2 (-2.9, 0.5)
WOMAC Function (0-68)	20.2 (11.0)	17.6 (8.9)	-2.5 (-6.2, 1.1)	21.4 (12.5)	14.0 (8.8)	-6.1 (-9.9, -2.3)*	-3.6 (-7.9, 0.8)
FFI-R Pain (25-100%)	41.0 (17.4)	38.4 (11.9)	-2.7 (-8.9, 3.5)	43.6 (16.2)	33.0 (9.3)	-7.0 (-13.5, -0.6)*	-4.3 (-12.0, 3.4)
FFI-R Stiffness (25-100%)	39.2 (15.7)	37.1 (13.7)	-2.1 (-7.3, 3.0)	42.8 (19.5)	32.3 (9.4)	-6.3 (-11.7, -0.9)*	-4.1 (-11.0, 2.7)
Stair climb (seconds)	5.32 (1.68)	5.35 (1.54)	0.04 (-0.55, 0.63)	6.57 (4.04)	5.58 (2.29)	-0.69 (-1.3, -0.08)*	-0.73 (-1.36, -0.11)*

* *p* < 0.05

Using a minimal clinically important improvement of 17% for WOMAC pain and 12% for WOMAC function [29], use of lateral wedges alone resulted in improved pain in 13 (54%) participants and improved function in 14 (58%) participants. When using the lateral wedges plus arch support, 14 (64%) participants had improved pain while 17 (77%) participants had improved physical function.

Wear times were similar between the two treatment periods, whether expressed as absolute or relative time values. The mean (sd) number of hours that participants self-reported insole wear was 150.7 (86.5) hours for the lateral wedges and 152.5 (121.0) hours for the lateral wedges plus arch support over the two-month intervention periods, which equated to 72.5 (19.2)% and 75.8 (24.4)% of total shoe wear time, respectively. There were no differences (*p* = 0.55) in self-reported comfort between the two conditions (lateral wedges = 6.4 (2.6) out of 10; lateral wedges plus arch support = 6.9 (2.5) out of 10). More participants indicated that they preferred their “first insole” (13/22) than their “second” (9/22), which translated into more individuals preferring the lateral wedges plus arch support (17/22; of these 17, 10 participants were randomized to the lateral wedges plus arch support first, while the other 7 received the lateral wedges plus arch support second). When asked to report overall change in symptoms compared to baseline, 15 individuals reported improvement with lateral wedges alone (7 = “much better”, 8 = “slightly better”) while 18 individuals reported improvement with the lateral wedges plus arch support (9 = “much better”, 9 = “slightly better”).

A total of 16 unique, yet minor, adverse events were self-reported during the study, 11 of which occurred during lateral wedges wear and five during lateral wedges plus arch support wear. With lateral wedges alone, the most common complaint was onset of foot pain or discomfort (*n* = 3), which lasted between one and three weeks. There were two reports of increased bunion pain, one lasting two weeks and the other for the duration of the intervention. Other complaints included: five weeks of toe cramping (*n* = 1), three weeks of lateral ankle pain (*n* = 1), one week of calf tightness (*n* = 1), generalized lower leg pain lasting three days (*n* = 1), and one report each of lower back and knee pain lasting one and ten weeks, respectively. During lateral wedges plus arch support wear, five individuals reported foot pain or discomfort ranging from two days to two weeks. Finally, three individuals underwent additional treatment during lateral wedges use (two instances of topical pain relief gel and one instance of foot and lower leg massage), while the same two treatments were completed by another two individuals during lateral wedges plus arch support wear.

## Discussion

Findings from this study indicate that the addition of custom foot arch support to 5-degree lateral wedges may result in clinical improvements in knee and foot symptoms, and timed stair climb, in people with medial knee OA and pronated feet. However these changes were generally not statistically significantly better than those observed with treatment by lateral wedges alone. Further, more participants subjectively preferred the supported lateral wedges overall, compared to lateral wedges alone. Data from this study provide important preliminary clinical information regarding safety and efficacy of combined insoles for the treatment of an important sub-group of patients with knee OA, namely those with concomitant pronated feet.

Although no other research has specifically evaluated effects of insoles in a subgroup of people with knee OA and concurrent pronated feet, our findings are consistent with the limited research investigating combined lateral wedge insoles with arch supports in heterogeneous samples of people with knee OA. Indeed, a previous uncontrolled study investigating a similar insole design showed improvements in pain and function in 42 individuals with medial knee OA and varus knee alignment. Skou et al showed that an insole that combined a custom-made arch support with an individualized amount of lateral wedging produced significant improvements in pain, function, and quality of life after an average of 7.75 months of wear [30]. Specifically, they found greater than 40% improvement, on average, in knee pain intensity (measured using a visual analog scale) with the combined insole. In the present study, a 30% improvement in WOMAC pain was observed with our combined insole, compared to only 12% with the lateral wedges alone. Similar findings have been reported elsewhere, with Jones et al showing more immediate improvement in knee pain with a combined insole compared to a standard lateral wedge alone in people with knee OA [21]. Taken together, these studies show the potential for greater improvement in knee pain with a combined insole than with a lateral wedge alone in people with knee OA.

Although the combined insole resulted in clinical improvements across all measured parameters, and we observed no significant effect on outcomes when participants were treated with lateral wedges alone, there was generally no statistically significant difference in outcomes when comparing between insole conditions. A larger sample size may have produced statistically significant differences, and results from this study can now be used to guide sample size calculations for future clinical trials. Importantly, though the lateral wedges plus arch support produced more symptomatic benefits over a two-month period, biomechanical data from the current cohort taken at the initial baseline assessment indicates that both insoles produced similar KAM reductions (albeit slightly larger reductions in the lateral wedges alone) compared to a no insole walking condition [24]. Thus, the differences in clinical outcomes in the present study cannot be attributed solely to the biomechanical effects of the lateral wedges. Indeed, a direct relationship between changes in KAM magnitudes and pain levels with foot-based interventions has not been shown in the literature [31, 32], suggesting that the relationship between measured external loads and knee pain magnitudes is complex. Rather, it is likely that the addition of the custom arch support played a role in reducing self-reported knee pain in the present study. The reasons for this are unclear, but may include indirect effects of the insoles, such as psychological factors on overall benefits based on insole preference or foot symptoms obtained with the combined insoles which then translated into perceived changes at the knee. Further research is needed to better elucidate the relationship between foot mechanics and knee symptoms in this patient population.

It was clear that participants preferred the lateral wedges with arch support. Indeed, 17 of 22 participants (78%) indicated an overall preference of that insole condition, and reported that the combined insole was slightly more comfortable under the feet. Any difference in comfort with the combined insole may partially explain why we observed clinical improvements with the combined insole but now with the lateral wedges alone. However, as there were no statistical differences when comparing group mean comfort ratings, any effect of perceived comfort would have occurred on an individual basis. Jones et al also reported slightly more perceived foot comfort with a combined insole compared to a lateral wedge alone, though the differences were also not statistically significant [21]. Given that 19/26 individuals reported at least some foot pain (values >25% on the FFI-R pain subscale) at the initial baseline assessment, assessment of changes in foot symptoms was important. Within-condition assessment of change in these symptoms from the current study would indicate that the lateral wedges plus arch support were able to improve foot symptoms. Finally, while not statistically tested, more self-reported adverse events were recorded during lateral wedges use (*n* = 11) than during the combined insoles (*n* = 5). While these reports were relatively minor and short-term, this finding provides further justification for the need to comprehensively assess the feet when providing any insoles treatment to people with knee OA to primarily target knee symptoms.

Our study is novel, and an important contribution to the literature for a number of reasons. First, this is the first study evaluating shoe-worn insoles in people with knee OA that has considered foot type as an inclusion or exclusion criterion. As noted above, given that pronated foot posture is common in people with knee OA [6], and is associated with a higher risk of developing knee pain and medial tibiofemoral cartilage damage [11], this subgroup in particular represents an important target for the study of shoe-worn insoles for the treatment of knee OA. More research with a homogeneous sample of pronated foot posture is warranted. This is also the first study, to our knowledge, to assess the impact of insoles on foot symptoms in people with knee OA. Since recent research has identified a link between foot symptoms and development of knee OA [9], and the fact that shoe-worn insoles evoke change directly at the feet, measurement of foot and ankle symptoms with foot-based treatment for knee OA is necessary. Indeed, given that more self-reported adverse events (especially lower leg, ankle, and foot issues) were reported in the present study with the lateral wedges alone, this finding provides important information necessary to inform clinical decision making in this particular subgroup. However, given the potential cost difference between the two types of insoles (potentially in excess of $300 per pair depending on the provider), a cost-effectiveness analysis of any symptomatic benefits of the combined insole must be conducted to assist in the justification of prescribing this addition to a standard lateral wedge.

There are some limitations to this study. First, although within-condition changes were observed, our relatively small sample size for this exploratory study likely impacted our ability to detect significant between-condition differences. In addition, certain limitations of a randomized crossover study in general (such as the potential for carryover and learning effects) must be considered. However our findings provide justification for larger studies in this area. Second, the two-month intervention was shorter than previous similar studies which utilized 6- or 12-month (or longer) intervention periods [33–35]. Although we did observe improvements in pain and function with the combined insole over this timeframe, it is unknown if these benefits would be maintained over the longer-term. Further, we relied on self-report data to examine outcomes such as usage and adverse events. Future research would benefit from more objective outcomes of wear time such as instrumented insoles that more accurately measure usage and would include some form of assessment of load during dynamic movement. Finally, more than half of our sample was comprised of individuals with mild radiographic disease severity. Recent research suggests that the relationship between biomechanics and knee symptoms in individuals with knee OA is stronger in more advanced radiographic disease [36], suggesting that shoe-worn insoles that attempt to evoke biomechanical changes at the knee may be less effective at improving knee symptoms in people with mild disease. Further research in samples with more advanced radiographic knee OA and pronated feet is required to determine the effectiveness of shoe-based intervention in this cohort.

## Conclusions

In conclusion, we found that the addition of custom arch support to standard lateral wedge shoe insoles resulted in improvements in foot pain and function in a group of individuals with knee OA and pronated feet. However changes in symptoms were not statistically different from those observed when participants were treated with lateral wedges alone. Given that shoe-worn insoles represent a relatively inexpensive treatment option with little patient burden, findings from the present study suggest further research is required to evaluate the role of combined insoles in managing knee and foot symptoms in these patients.

## Abbreviations

EVA: Ethyl-vinyl-acetate
FFI-R: Foot function index revised version
FPI: Foot posture index
KAM: Knee adduction moment
KL: Kellgren and Lawrence
NRS: Numerical rating scale
OA: Osteoarthritis
WOMAC: Western Ontario and McMaster Universities Osteoarthritis Index
